# The *Non-Coding RNA* Journal Club: Highlights on Recent Papers—7

**DOI:** 10.3390/ncrna5020040

**Published:** 2019-06-11

**Authors:** Hua Xiao, Patrick K. T. Shiu, Marta Gabryleska, Simon J. Conn, Abhishek Dey, Kausik Chakrabarti, Manuel Regouc, Martin Pichler, Ulf Andersson Vang Ørom, Gaetano Santulli, Satoshi Nishiwada, Ajay Goel, Vaishnavi Nagarajan, Lisa Timmons, Suresh K. Alahari, Noemi Laprovitera, Manuela Ferracin, Po Hu, Hailing Jin

**Affiliations:** 1 Division of Biological Sciences, University of Missouri, Columbia, MO 65211, USA; xiaohu@missouri.edu; 2 Human Genetics Unit, MRC, IGMM, University of Edinburgh, Edinburgh EH4 2XU, UK; mgabryel@staffmail.ed.ac.uk or marta.gabryelska@flinders.edu.au; 3 Flinders Centre for Innovation in Cancer, Flinders University, Adelaide, SA 5042, Australia; 4 Department of Biological Sciences, The University of North Carolina at Charlotte, 9201 University City Boulevard, Charlotte, NC 28223, USA; adey2@uncc.edu; 5 Division of Oncology, Department of Internal Medicine, Medical University of Graz, 8036 Graz, Austria; manuel.regouc@stud.medunigraz.at; 6 Research Unit for Non-coding RNAs and Genome Editing, Medical University of Graz, 8010 Graz, Austria; 7 Department of Molecular Biology and Genetics, Aarhus University, 8000 Aarhus C, Denmark; 8 Einstein College of Medicine–Montefiore University Hospital, New York, NY 10461, USA; 9 Center for Gastrointestinal Research, Baylor Scott & White Research Institute and Charles A. Sammons Cancer Center, Baylor University Medical Center, Dallas, TX 75246, USA; Satoshi.Nishiwada@BSWHealth.org; 10 Beckman Research Institute at City of Hope Comprehensive Cancer Center, 5th Avenue, Monrovia, CA 91016, USA; 11 Department of Molecular Biosciences, The University of Kansas, Lawrence, KS 66045, USA; v612n423@ku.edu; 12 Department of Biochemistry and Molecular Biology, LSUHSC School of Medicine, New Orleans, LA 70112, USA; 13 Department of Experimental, Diagnostic and Specialty Medicine (DIMES), University of Bologna, 40138 Bologna, Italy; noemi.laprovitera@unibo.it; 14 Department of Microbiology & Plant Pathology, Center for Plant Cell Biology, Institute for Integrative Genome Biology, University of California, 900 University Ave., Riverside, CA 92521, USA; pohup@ucr.edu

## 1. Introduction

We are delighted to share with you our seventh Journal Club and highlight some of the most interesting papers published recently. We hope to keep you up-to-date with non-coding RNA research works that are outside your study area. The *Non-Coding RNA* Scientific Board wishes you an exciting and fruitful read.

## 2. Epigenetic Silencing of a Triplet-Expanded Gene

### Highlight by Hua Xiao and Patrick K. T. Shiu

Although it is known that trinucleotide repeat expansions cause a number of neuromuscular disorders (e.g., Friedreich’s ataxia or FRDA), the exact mechanisms through which gene functions are affected remain uncertain for most of these diseases. In a recent issue of *Cell*, Eimer et al. showed that an intronic triplet expansion can trigger the biogenesis of short interfering RNAs (siRNAs), leading to the transcriptional downregulation of the afflicted gene [1].

Previously, Sureshkumar Balasubramanian’s group showed that an intronic GAA/TTC repeat expansion at the *Arabidopsis ISOPROPYL MALATE ISOMERASE LARGE SUBUNIT 1* (*IIL1*) gene leads to its downregulation and a growth defect at elevated temperatures. Here, they demonstrated that this downregulation is associated with a transcriptionally repressed chromatin state characterized by an abundance of histone H3 lysine 27 trimethylation (H3K27me3) marks. Apparently, the triplet expansion leads to the production of double-stranded RNAs, which are converted to siRNAs by DICER-LIKE 3 (DCL3). The siRNAs, through the RNA-dependent DNA methylation (RdDM) pathway and the polycomb repressive complex (PRC), direct the repressive epigenetic modification at the *IIL1* locus.

These findings suggest that siRNA-mediated silencing could be a possible mechanism for diseases such as FRDA. Future studies will shed light on whether the underlying mechanisms of certain repeat expansion disorders are conserved across systems and/or kingdoms.

## 3. The RNA Interactomics X-Files

### Highlight by Marta Gabryleska and Simon J. Conn

RNA molecules lead fascinating lives, and from the beginning until the end, they encounter many interacting partners. We have entered the era of RNA interactomics, and new technologies are necessary to profile these interactions toward illuminating their physiological relevance. The XRNAX (protein-crosslinked RNA extraction) method, recently published in *Cell* [2], expands our understanding of the interactions between RNA and protein and, in particular, non-coding RNAs and their bound proteome at previously unachievable resolution.

Similar to plethora techniques for mapping RNA–protein interactions, XRNAX utilizes UV irradiation to crosslink protein and RNA, in vivo, with TRIzol extraction performed to harvest the complexes. However, unlike other approaches, the authors collect the interphase between the aqueous and non-aqueous phases, which are usually carefully omitted in the standard TRIzol extraction protocol, and enrich the desired RNA–protein complexes. The high-throughput mass spectrometric and next-generation sequencing analyses of these complexes delineated both the interacting proteome and transcriptome, respectively, with such resolution as to identify a novel RNA binding motif. Uniquely, XRNAX achieved the first identification of the human non-coding RNA binding proteome, with over 700 proteins identified, including BRCA1 and confirmed interactions for TP53, which was previously a controversial interacting partner for non-coding RNA.

XRNAX is the newest member of a family of RNA interactomics techniques, offering great potential to uncover the secrets of the complex lives of non-coding RNAs.

## 4. Mechanistic Model of Telomerase Ribonucleoprotein Enzyme

### Highlight by Abhishek Dey and Kausik Chakrabarti

Telomerase is a ribonucleoprotein complex that extends the chromosomal termini known as the “telomere” by adding DNA repeats and thus neutralizing the continuous shortening of DNA due to incomplete DNA replication. The telomeric repeat addition processivity ascends from the species-specific usage of telomerase RNA (TR) template domain, while the catalytic activity is mediated by telomerase reverse transcriptase (TERT) activity. However, in the absence of a three-dimensional model of telomerase, the exact mechanism of telomerase-regulated telomere lengthening remained elusive.

Recent cryo-Electron Microscopy (EM) models of both human [3] and Tetrahymena [4] telomerase holoenzyme complexes propose important information about the key RNA–protein interactions. Both models depict TR as a bilobal structure. One end of this structure, which is also called the “catalytic lobe”, displays TERT as a ring, encircling the template-ssDNA substrate with the template-pseudoknot of TR wrapping around the ring. The second lobe of the TR interacts with various species-specific accessory proteins that are essential for telomerase maturation, biogenesis, and their recruitment to the telomere. Indeed, the RNA–protein interactions within this lobe are vital for the holoenzyme activity since mutations within this region in humans are known to cause several diseases [3].

These models have improved our understanding about the mechanistic properties of the telomerase holoenzyme and should provide paths to explore structure-function relationship of this important ribonucleoprotein complex for developing new therapeutics.

## 5. Regulatory Functions of lncRNA MALAT1 in Breast Cancer

### Highlight by Manuel Regouc and Martin Pichler

The dysregulated expression of long non-coding RNAs (lncRNAs) influences the development of many different cancer types. In a recently published study, Kim et al. have shown that an overexpression of lncRNA MALAT1 (metastasis-associated lung adenocarcinoma transcript 1) promotes breast cancer metastasis. Knockout of the *MALAT1* gene in MMTV-PyMT (mouse mammary tumor virus-polyomavirus middle T antigen) mice leads to a higher existence of circulating tumor cells (CTC) and causes an upregulation of tumor-promoting transcription factors such as TEAD1 (TEA domain family member 1). They demonstrated that MALAT1 inhibits the interaction between TEAD1 and its cofactor YAP (yes-associated protein 1) by blocking the transactivation domain of TEAD1. In further consequence, MALAT is able to downregulate metastasis, promoting proteins such as integrin β4 (ITGB4) and vascular endothelial growth factor (VEGFA) [5].

The results of Kim et al. are in strong contrast to other related publications due to their remarkable model systems and experiments. The function of MALAT1 as a tumor-suppressing factor refutes the previous hypotheses and shows the tasks of lncRNAs from a different angle. The new insights into the regulatory mechanisms of lncRNAs may allow new therapeutic options for breast cancer patients in the future.

## 6. Splicing of Long Non-Coding RNAs

### Highlight by Ulf Andersson Vang Ørom

Long non-coding RNA (ncRNA) transcripts have several of the same features as protein-coding mRNA, yet long ncRNAs are often less efficiently spliced than mRNAs. This observation has fueled debates on whether splicing controls the function and localization of long ncRNAs. Krchnáková et al. have addressed this important question using ncRNA-a2 [6], an activating long ncRNA, as a model. The authors rigorously analyzed the features of the long ncRNA transcript and found that features such as the secondary structure and splicing inhibitory sequences are not responsible for this difference between long ncRNA and mRNA splicing. The authors show that one distinguishing feature is that long ncRNAs have longer exons and introns than mRNAs, which have been shown to affect the splicing efficiency.

By a more specific analysis of splice-site sequences, the authors showed a positive correlation between the strength of the 5′ splice-site and the polypyrimidine tract and long ncRNA splicing efficiency. The authors provide evidence that long ncRNAs are more dependent on basic splice-site sequences than mRNAs due to the decreased productive binding of SR proteins to long ncRNAs.

In addition, the authors reported that removing the intron from ncRNA-a2 does not affect its enhancer-like function, suggesting that splicing is not essential for function for at least a group of long ncRNAs.

## 7. Exosomal MyomiRs Mediate Long-Distance Calls between Heart and Bone Marrow

### Highlight by Gaetano Santulli

Communication is a key feature in cardiovascular biology, and cells in multicellular organisms communicate with each other via a number of mechanisms, including direct cell–cell contact, cell–matrix interaction, long-range signals, electrical signals, and extracellular chemical molecules. In this sense, extracellular vesicles and exosomes represent an emerging field of investigation. Cheng et al. demonstrated that myocardial microRNAs (myomiRs) carried in circulating exosomes (cardiosomes) allow a systemic response to cardiac injury [7]. The authors elegantly show that following myocardial infarction cardiosomes mediates the transfer of specific myomiRs to mononuclear cells within the bone marrow, where they target CXC chemokine receptor 4 (CXCR4), causing its downregulation. This discovery has major implications in the clinical scenario, since targeting cardiosomal miRs may provide a novel therapeutic approach for the treatment of ischemic heart disease.

## 8. A Liquid Biopsy Biomarker for Predicting Response to Chemotherapy in Pancreatic Ductal Adenocarcinoma: A Significant Step Forward in Precision Medicine

### Highlight by Satoshi Nishiwada and Ajay Goel

Pancreatic ductal adenocarcinoma (PDAC) is one of the most lethal human cancers, and the majority of patients present with advanced disease, when the disease is mostly unresectable. In PDAC patients, the first-line treatment consists of a combination regimen of 5-fluorouracil, oxaliplatin, irinotecan, and leucovorin (FOLFIRINOX), or gemcitabine (GEM) plus nanoparticle albumin-bound paclitaxel (nab-paclitaxel). However, unfortunately, only a very small subset of patients respond to such treatments, and an appropriate selection of patients who might benefit from such treatment modalities requires the availability of biomarkers that can guide decision-making, which is currently lacking. In a recent issue of *Annals of Surgery*, Meijer et al. provided very exciting evidence that plasma levels of miR-181a-5p might actually offer a promising biomarker potential that can predict the response to FOLFIRINOX in patients with pancreatic cancer [8].

In this study, the authors used a microarray-based profiling approach to discover deregulated miRNAs, in both pre-chemotherapy and post-chemotherapy plasma specimens, in PDAC patients. Based upon their progression-free survival (PFS) following FOLFIRINOX treatment, the investigators identified a panel of nine candidate plasma miRNAs that could predict the response to this first-line therapy. Thereafter, the expression of these nine miRNAs was validated in an independent patient cohort, which led to the identification of the most significant miRNA biomarker: miR-181a-5p. The plasma levels of miR-181a-5p were significantly downregulated in patients with non-progressive disease following FOLFIRINOX therapy. Furthermore, in multivariate analysis, a combination model comprising of miR-181a-5p expression and CA19-9 levels significantly correlated with improved PFS and overall survival. To add another layer of specificity, the authors were also able to confirm the correlation between the expression of this miRNA between tissue and plasma specimens, highlighting its potential as a non-invasive biomarker.

These results are truly exciting, as these provide a glimpse into the potential of miRNAs, especially as non-invasive, liquid biopsy biomarkers, for making a significant clinical impact for appropriate patient selection, which is an important step forward as we usher into the era of precision medicine for patients suffering from a lethal malignancy such as pancreatic cancer.

## 9. Translation of an ALS/FTD-Associated Hexanucleotide Repeat Expansion: Nuclear Impairment, dsRNA Accumulation and Neuronal Toxicity

### Highlight by Vaishnavi Nagarajan and Lisa Timmons

A GGGGCC hexanucleotide repeat expansion in the C9orf72 gene, encoding a likely guanine nucleotide exchange factor, was uncovered in 2011 as an associative allele for hereditary amyotrophic lateral sclerosis and frontotemporal dementia (ALS/FTD). C9orf72 repeats lie within a first intron and can be retained in transcripts. Repeat-associated RNAs from both sense and antisense strands are observed in patient-derived cells, with some experimental evidence for their translation. Thus, peptides from either strand are possible, with poly-PR dipeptides produced from antisense RNAs.

A 15th February paper published in *Science* [9] evaluated the in vivo consequences of poly-PR expression in developing mice. The effects were lethal, with brain atrophy and neuronal loss observed in surviving mice. Poly-PR localized to the nucleus, associated with heterochromatin, and led to the loss of heterochromatin protein 1α. Notably, an increase in the RNA expression of repetitive elements was observed, along with the accumulation of dsRNA, as revealed by increased staining using dsRNA-specific antibodies. The loss of HP1α also correlated with the presence of active caspase-3. Taken together, these results allow for an intriguing model in which the translation of hexanucleotides repeats leads to chromatin alterations, dsRNA production, and eventually apoptosis.

The developmental context of the experiments may help capture snapshots of progressive neuroinflammation, leading to cell death—a feat that is not possible using post-mortem tissue. Future experiments will help determine whether this largely transgene-based approach is germane to the molecular pathology associated with human disease.

## 10. *EPIC1* Identified as an Oncogenic lncRNA That Interacts with MYC and Promotes Cell Cycle Progression

### Highlight by Suresh K. Alahari, Noemi Laprovitera and Manuela Ferracin

Long non-coding RNAs (lncRNA) play an important role in tumorigenesis. Epigenetic alterations have been established as one of the hallmarks of tumorigenesis, but their involvement on lncRNA gene regulation was still unclear. In a recent issue of *Cancer Cell* [10], Wang et al. developed an analysis pipeline to characterize the DNA methylation landscape of lncRNA genes across 33 cancer types using two large-scale epigenetic datasets (The Cancer Genome Atlas, TCGA and The Cancer Cell Line Encyclopedia, CCLE projects). They observed that, contrary to protein-coding genes, lncRNAs are epigenetically activated in tumors through the hypomethylation of their promoter regions. In their analysis, Wang et al. demonstrated that the epigenetic activation of lncRNAs was associated with the co-occurrence of TP53 mutation and worse survival.

Epigenetically induced lncRNA (*EPIC1*) has been identified as the most epigenetically activated lncRNA in several cancers. Breast cancer patients with *EPIC1* hypomethylation and increased *EPIC1* expression have the worst survival. Further analysis indicated that *EPIC1* epigenetic activation associated with luminal B and Her2 subtypes of breast cancer. *EPIC1* has been shown to promote cell proliferation, anchorage independent growth, cell cycle progression in breast cancer, and in vivo xenograft tumor growth, suggesting the oncogenic function of *EPIC1*, and thus could be a good target for breast cancer treatment. RNA sequencing and other molecular analysis revealed that oncogenic function of *EPIC1* is through the association with the MYC protein. The authors demonstrated that *EPIC1* interacts in the nucleus with the 148–220 aa region of MYC through its 129–283 nt region and specifically regulates MYC’s occupancy on a subset of MYC targets, enhancing its heterodimerization with MAX and its activity as transcription factor.

These findings suggest that DNA methylation regulates the lncRNAs involvement in cancer progression, and *EPIC1* will be a good prognostic marker and a good therapeutic target to develop novel therapies for breast cancer.

## 11. Spliceosomal Intron RNAs Promote Cell Survival

### Highlight by Po Hu and Hailing Jin

Pre-mRNA molecules often contain intron and exon sequences in all known eukaryotic genomes. Introns, the non-coding component of pre-mRNA, are excised from mature mRNA and usually debranched and degraded rapidly. However, in a recent *Nature* issue, David Bartel’s group challenged this view by demonstrating that the spliceosomal introns help yeast cells survive starvation. They think that 34 specific excised introns in *Saccharomyces cerevisiae* become stabilized and accumulate during the stationary phase to adjust cell growth to adapt to starvation conditions or other stress conditions.

Previously, Morgan et al. performed RNA-seq analysis on two *S. cerevisiae* samples: one in log-phase growth, and the other cultured in a nutrient-deficient medium. Some excised linear introns (including ECM33) were found to accumulate in the saturated status, which were then identified as “stable introns” [11]. ECM33 intronic RNA with the MS2 hairpin was pulled down and the intron lariat spliceosome (ILS) complex was identified to protect stable introns in saturated culture. Through the inspection of RNA-seq reads, two characteristics of stable introns were defined: (1) the short distance between the lariat branch point and 3′ splice site, and (2) expression within a cellular context in which introns are stabilized. All the non-stable introns tested became stable when they were modified to fit these two criteria. Furthermore, by using rapamycin, the inhibitor of TORC1 (target of rapamycin complex 1), they found that stable introns function within the TORC1-mediated stress response in yeast: TORC1 inhibits stable intron formation, as stable introns inhibit yeast cell growth.

In the same issue, a companion paper from Sherif Abou Elela’s group also demonstrated the surprising function of introns on cell response to starvation in yeast [12]. However, Parenteau et al. identified different intron forms: unspliced transcripts. Together, these two papers provide a new unexpected function of introns within eukaryotes. The intriguing role of introns in yeast starvation response generates an exciting starting point for future research prospects.
